# Targeting heterogeneity of adrenocortical carcinoma: Evaluation and extension of preclinical tumor models to improve clinical translation

**DOI:** 10.18632/oncotarget.12685

**Published:** 2016-10-15

**Authors:** Constanze Hantel, Igor Shapiro, Giada Poli, Costanza Chiapponi, Martin Bidlingmaier, Martin Reincke, Michaela Luconi, Sara Jung, Felix Beuschlein

**Affiliations:** ^1^ Endocrine Research Unit, Medizinische Klinik und Poliklinik IV, Ludwig-Maximilians-Universität, Munich, Germany; ^2^ Department of Surgery, Ludwig-Maximilians-Universität, Munich, Germany; ^3^ Endocrinology Unit, Department of Experimental and Clinical Biomedical Sciences, University of Florence, Florence, Italy

**Keywords:** adrenocortical carcinoma, patient-derived tumor-xenograft, preclinical animal model, cell line, clinical translation, Abbreviations: ACC: adrenocortical carcinoma, STR: short tandem repeat, PDTX: patient derived tumor xenografts

## Abstract

In recent years it has been recognized that clinical translation of novel therapeutic strategies for patients with adrenocortical carcinoma (ACC) often fails. These disappointing results indicate that the currently utilized tumor models only poorly reflect relevant pathophysiology and, thereby, do not predict clinical applicability of novel pharmacological approaches. However, also the development of new preclinical ACC models has remained a challenge with only one human cell line (NCI-H295R) and one recently established human pediatric xenograft model (SJ-ACC3) being available for this highly heterogeneous malignancy. Our current study furthermore reveals a poor reproducibility of therapeutic action between different clones of the most commonly used tumor model NCI-H295R. In an attempt to broaden the current preclinical armamentarium, we aimed at the development of patient-individual tumor models. During these studies, one xenograft (MUC-1) displayed marked engraftment and sustained tumor growth. MUC-1 tumor analysis revealed highly vascularized, proliferating and SF-1 positive xenografts. In a next step, we characterized all currently available human tumor models for ACC for Ki67, SF-1 and EGF-receptor status in comparison with MUC-1-xenografts. In addition, we established a primary culture, which is now viable over 31 passages with sustained nuclear SF-1 and cytoplasmic 3βHSD immuno-positivity. Subsequent investigation of therapeutic responsiveness upon treatment with the current systemic gold standard EDP-M (etoposide, doxorubicin, cisplatin and mitotane) demonstrated maintenance of the clinically observed drug resistance for MUC-1 exclusively. In summary, we provide evidence for a novel patient-derived tumor model with the potential to improve clinical prediction of novel therapeutic strategies for patients with ACC.

## INTRODUCTION

Adrenocortical carcinomas (ACC) are rare and highly malignant tumors with a poor prognosis. In recent years genetic and molecular profiling of surgical tumor specimens have led to the identification of novel biomarkers for ACC with potential prognostic impact [1]. However, the exact contribution of underlying pathways for adrenal tumorigenesis and their relevance for individualized therapeutic decisions is still largely unknown. In recent years new targeted therapies have been successfully introduced for various cancer types, but initial clinical evaluation of such therapies in patients with ACC have been disappointing [2–4]. Consequently, the classical multi-chemotherapeutic EDP-M scheme consisting of etoposide, doxorubicin, cisplatin and mitotane remains the gold standard therapy for patients not amendable for surgical resection [5]. Despite this therapeutic option, the overall survival of patients is still poor. Moreover, dose limiting site effects and therapy associated severe adverse events are commonly encountered [5]. Thus, novel treatment options for patients with extended disease are urgently needed.

Tumor models are important tools for preclinical therapeutic studies, but translation of novel therapeutic strategies in patients often fails indicating that prediction of clinical success solely on the basis of currently utilized models is not reliable. One reason for this shortcoming is that only two human cell lines, NCI-H295R and SW-13, are available for ACC, which do not reflect tumor heterogeneity and specific therapeutic responses of individual patients [6, 7]. In addition, SW-13 had originally been established from surgical material of a small cell carcinoma of the adrenal gland [7]. Thereby, its adrenocortical origin has been repeatedly questioned. Due to the lack of other ACC tumor models SW-13 cells have been used to complement preclinical experiments, but its predictive clinical value is highly debated as it lacks features of adrenocortical differentiation.

Even though these two commonly available cell lines can be utilized as xenografts in immunodeficient mice [8, 9], they originate from cell suspensions following long-term *in vitro* culture. There is good evidence that selection during multiple cell culture passages grossly changes biological properties compared to the original patient tumor [10]. To overcome this limitation, patient-derived tumor xenografts (PDTX) engrafted in immunodeficient mice have been established and tested for a variety of cancer types [11]. Following the same approach the establishment of a novel tissue-based xenograft model for pediatric ACC (SJ-ACC3) was recently reported [12]. Unfortunately, no cell line for complementary *in vitro* experiments could yet be derived from this xenograft.

Here we report on the limitations in clinical prediction of classical human tumor models for ACC as well as on the establishment of a new tumor model with the potential to improve the current unsatisfactory situation.

## RESULTS

### Clone dependent functional heterogeneity of NCI-H295R derived xenografts

During recent *in vivo* experiments on novel chemotherapeutic treatment schemes for ACC, our workgroup incidentally detected marked differences during tumor development of two clones of NCI-H295R (denoted as clone 1 and clone 2). While clone 1 was originally obtained from ATCC and utilized over a long period in our laboratory, clone 2 was again purchased from ATCC in 2012. Of note, both clones were recently analyzed by short-tandem repeat analyses confirming their authentication as NCI-H295R cells.

**Figure 1:**
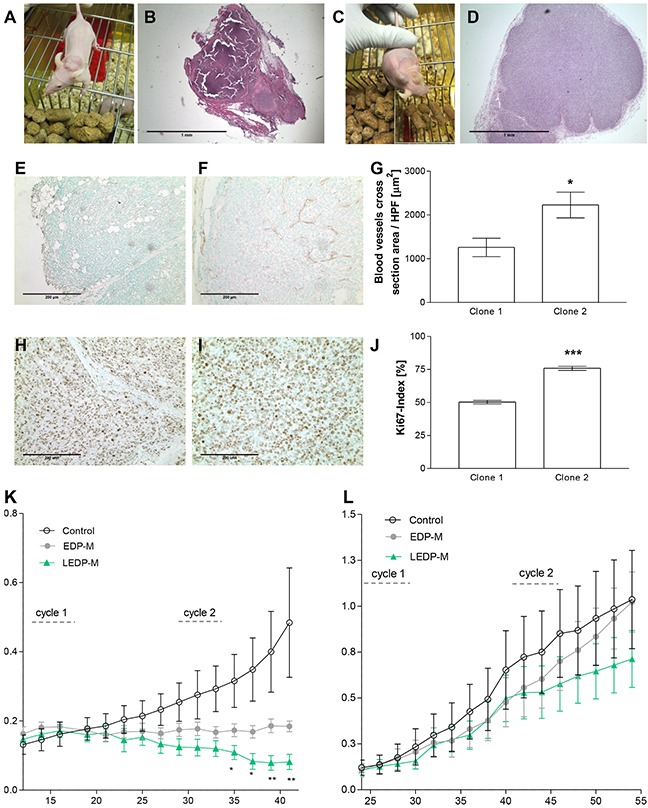
Pictures of athymic nude mice bearing NCI-H295R xenografts and H&E sections of clones 1 **A, B.** and 2 **C, D.** Representative CD31 **E, F.** and Ki67 **H, I.** stainings as well as the quantification of blood vessels cross sections **G.** and proliferation index **J.** of clone 1 and 2 tumors. Effects on tumor size of NCI-H295R xenografts of clone 1 **K.** and clone 2 **L.** after two therapeutic cycles with EDP-M (etoposide, doxorubicin, cisplatin and mitotane) and LEDP-M (etoposide, liposomal doxorubicin, liposomal cisplatin and mitotane). Stars denote significant differences compared with EDP-M.

Macroscopically, xenografts derived from clone 2 (Figure 1C) developed large blood vessels, while this phenomenon was not noticed for clone 1 (Figure 1A). Increased tumor vascularization was confirmed by a detected higher number of blood vessel cross sections [μm^2^] of CD31 stained tumor slides (clone 1: 1258.6±209.1 vs. clone 2: 2228.5±293.7; p<0.05; Figure 1G). Moreover, we observed less effective engraftment of clone 2 in comparison to clone 1. Furthermore, subsequent histological and immunohistochemical analyses revealed highly necrotic xenografts of clone 1, while the proliferation rate was significantly higher for tumors derived from clone 2 (75.8±1.6%) compared to clone 1 (50.3±1.3%;p<0.001; Figure 1J). Interestingly, similarly extensive heterogeneities of *in vivo* properties were noticed independently by two different european laboratories (munich workgroup clones 1 and 2 in Figure 1, and Florence workgroup clones 3 and 4 in Supplementary Figure S1).

To investigate whether these differences in biological behavior might have an impact on therapeutic prediction, we performed an intervention study with xenografts of clone 2 including the clinical gold standard treatment for ACC (etoposide, doxorubicin, cisplatin and mitotane, EDP-M) as well as a novel liposomal variant LEDP-M (etoposide, liposomal doxorubicin, liposomal cisplatin and mitotane). Previous studies with clone 1 had revealed significant differences between controls and LEDPM-treated tumors. Moreover, we detected significantly reduced tumor sizes in LEDP-M-treated tumors in comparison with EDP-M-treated tumors following the second therapeutic cycle as depicted by stars (Figure 1K, [13]). Analogous chemotherapeutic treatments on xenografts obtained from clone 2 did not reveal any significant reduction in tumor sizes upon EDP-M or LEDP-M treatment (Figure 1L). Thus, this comparative study revealed a marked and relevant spread of results based on the classical and most commonly used ACC tumor model.

### Establishment and characterization of MUC-1 xenografts

In an attempt, to establish patient-individual endocrine tumor models, our working group initiated in a next step the implantation of ACC derived patient tumor specimen. During these studies, one xenograft (MUC-1) showed particular engraftment properties and sustained tumor growth. The respective tumor tissue was obtained from a 24-year-old male patient with a primary diagnosis of a left adrenal mass of 22 cm. While the tumor was initially diagnosed because of abdominal discomfort, no clinical symptoms of overt Cushing syndrome were reported. However, urinary steroid metabolome analysis [14] revealed a malignant secretory profile indicative of hormonal activity. The patient underwent adrenalectomy, nephrectomy and lymphadenectomy, and pathological examination revealed a Ki67 index of 30-40%. Despite extended radical resection, the patient developed abdominal metastatic spread, which was treated with four cycles of etoposide, doxorubicin, cisplatin and mitotane. The tumor pieces utilized for the preclinical study were derived from a subsequently developed subcutaneous metastasis at the neck.

### Characterization of MUC-1 xenografts

**Figure 2:**
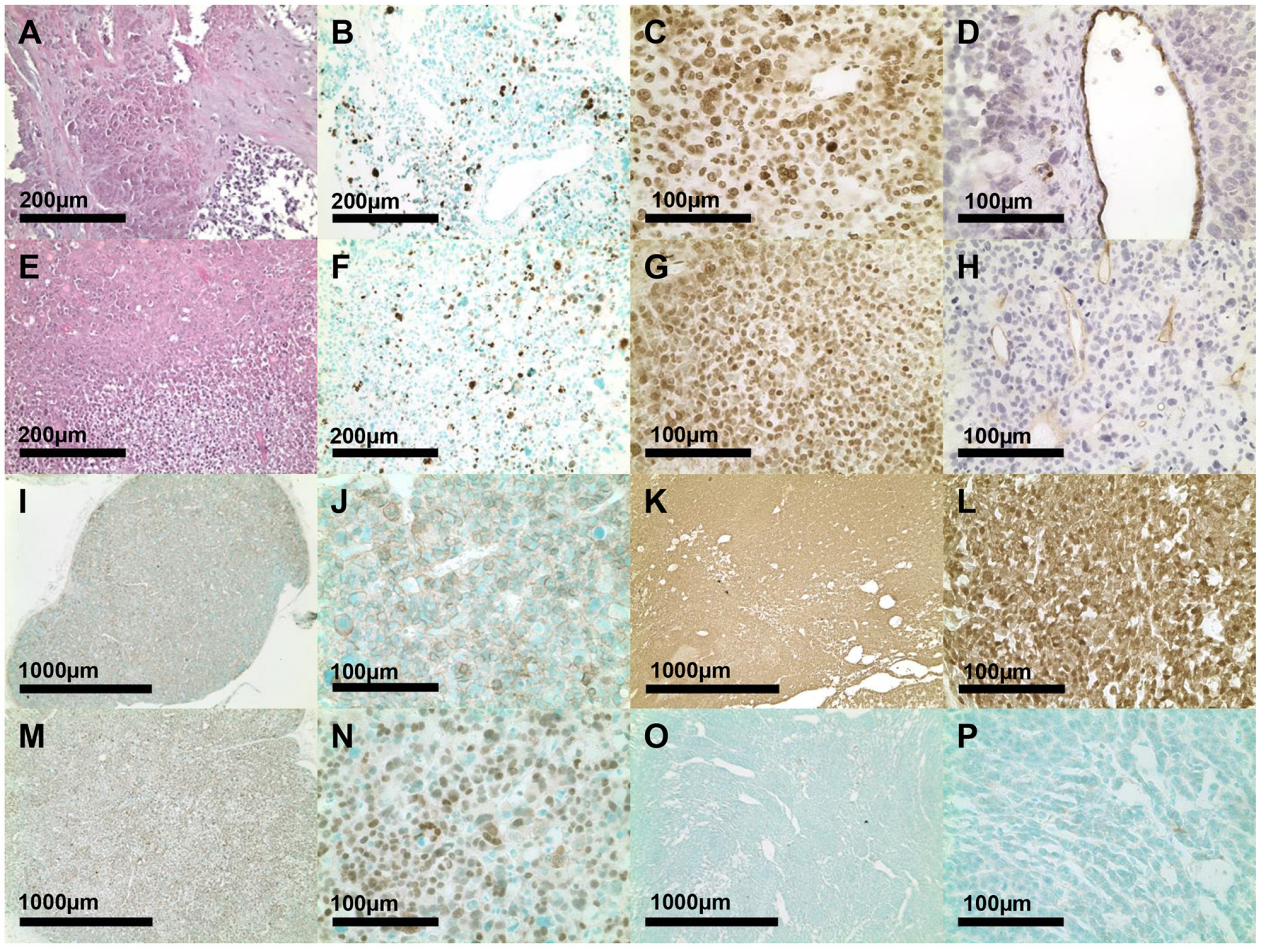
H&E, Ki67, SF-1 and CD-31 tumor analysis from the original patient tumor **A-D.** and of MUC-1 xenograft **E-H.** derived tumor slides from passage 2. Immunohistochemical β-catenin **I-L.** and p53 stainings **M-P.** of MUC-1 (I, J. M, N) and NCI-H295R (K, L, O, P) tumor slides.

Upon tumor inoculation of ten athymic nude mice aggressive tumor growth of two of the xenografts was noted. Immunohistochemical analyses revealed a highly vascularized and SF-1 positive (27.8 ± 1.6 cells/high power field)) xenograft in the murine host (Figure 2A–2D). The Ki67 index (9.5 ± 0.4 %) was comparable with the original surgical sample (p = n.s.; Figure 2E-H). Moreover, immunohistochemical analysis revealed β-catenin and p53 positivity for MUC-1 xenografts (Figure 2I-L) with staining patterns different from that obtained for the classical NCI-H295R tumor model (Figure 2M-P).

**Figure 3:**
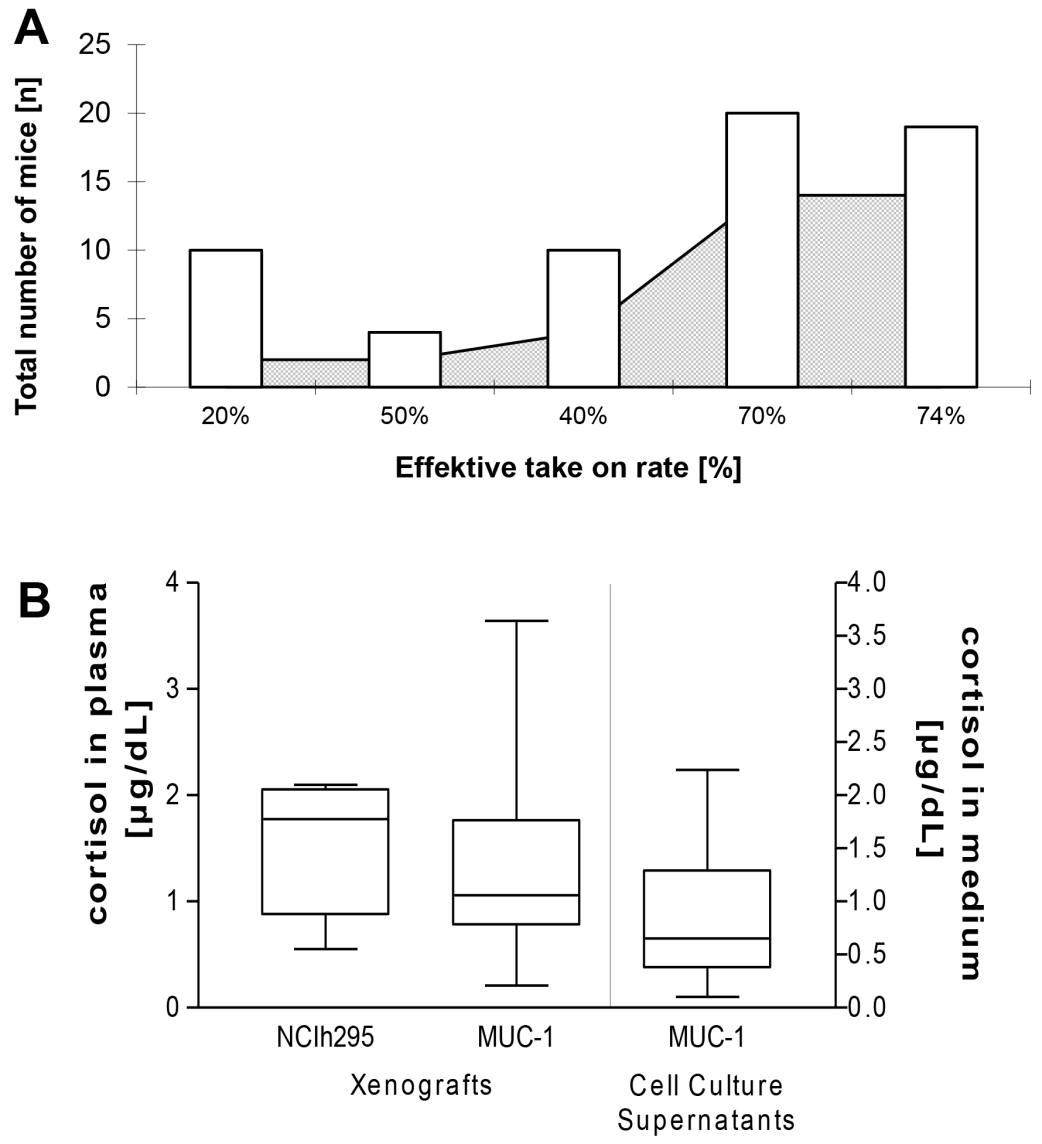
Schematic illustration of increasing engraftment rates (**A**, the white bars represent the total number of implanted mice per each passage while the grey area depicts the effective take on rates) Cortisol measurements of plasma from NCI-H295R (n=6) and MUC-1 (n=17) tumor-bearing mice *in vivo* and of cell culture supernatants (n=14 of passages 3, 4, 5, 6, 8, 9, 10, 12 and 15 which were offset against a medium blank in triplicate) of MUC-1 cells *in vitro* **B.**

The established xenograft was passed over into another group of animals. This procedure was repeated several times up to passage 5 (n= 10 (P1), 4 (P2), 10 (P3), 20 (P4) and 19 (P5), respectively) with sustained tumor growth. Remarkably, with the number of passages also the effective take-on rate increased from 20% in passage 1 up to approximately 70% in passages 4 and 5 (represented as number of implants showing large increase in tumor size per number of total implanted in %; Figure 3A). Simultaneously, the effective take-on time (defined as time until the first tumor of the passage reached a tumor size of 1.5 cm) decreased from 198 days in passage 2 down to 71 days in passage 5.

### Comparison of MUC-1 with commonly available ACC xenografts

**Figure 4:**
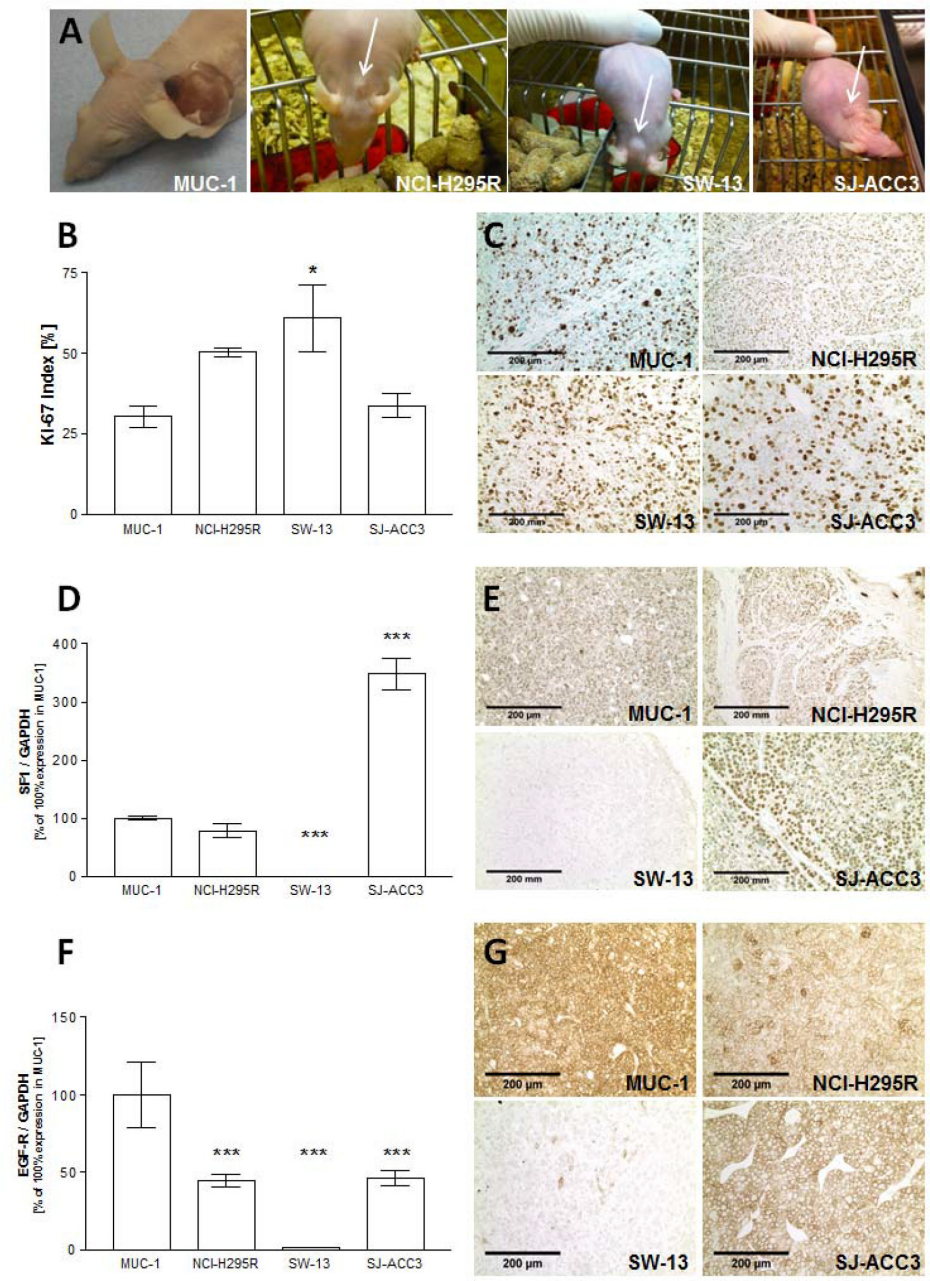
Pictures from NCI-H295R, SW-13, SJ-ACC3 and MUC-1 xenografts in athymic nude mice **A.** Quantification of Ki67indices **B.** as well as representative immunohistochemical stainings from all tumor models **C.** Real-Time PCR analysis and immunohistochemical stainings of SF-1 **D, E.** and EGF-receptor **F, G.** for all tumor xenografts. Stars represent significance vs. MUC-1 (*, p<0.05; **, p<0.01; ***, p<0.001).

We further investigated the Ki67 indices [%] of all commonly available xenograft models for ACC by immunohistochemistry. While SW-13 tumors showed significantly higher proliferation rates (60.8±10.3%, p<0.05 versus MUC-1), Ki67 indices of NCI-H295R (50.3±1.3%) and SJ-ACC3 (33.7±3.6%) were comparable to those of MUC-1 xenografts (30.3±3.3%; p= n.s, Figures 4B and 4C).

In a next step, we analyzed SF-1 RNA (Figure 4D) and protein (Figure 4E) levels in all currently available ACC tumor models by quantitative real time PCR and immunohistochemistry, respectively. While SF-1 expression in MUC-1 (100 ± 0.0%) and NCI-H295R (78.3± 12.3%) was comparable (p=n.s.), we detected a highly significant SF-1 overexpression in the pediatric ACC tumor model SJ-ACC3 (348.2± 27.4%, p < 0.001 vs. MUC-1) and almost no detectable SF-1 expression in SW-13 xenografts (0.03± 0.0%, p < 0.001vs. MUC-1). Immunohistochemical analyses of representative tumor slides confirmed these findings (Figure 4E).

Regarding hormonal status, ELISA measurement revealed cortisol levels of 1.11 ± 0.2 in plasma samples of MUC-1 tumor bearing mice (n=17) which were statistically comparable with cortisol levels measured in plasma samples of mice bearing the commonly utilized hormonal active NCI-H295R-xenografts (1.57 ± 0.2; p = 0.5, n=6; Figure 3B).

In addition, we choose the EGF-receptor as one example to characterize potential differences in cellular signaling pathways between MUC-1 and the other currently existing *in vivo* models. While EGF-receptor expression was almost abrogated in the SW-13 tumor model (1.2 ± 0.2%; p < 0.001 vs. MUC-1) a significant higher expression was detectable in MUC-1 tumors (100 ± 0%) compared with all other tumor models (NCI-H295R: 44.5 ± 4.1% and SJACC-3: 45.9 ± 4.9%; both p < 0.001vs. MUC-1; (Figure 4F). Immunohistochemical analysis in tumor slides of the different xenografts also confirmed these as well as membranous receptor staining specifically for MUC-1, NCI-H295R and SJ-ACC3 xenografts (Figure 4G).

### Establishment of a MUC-1 cell line

**Figure 5:**
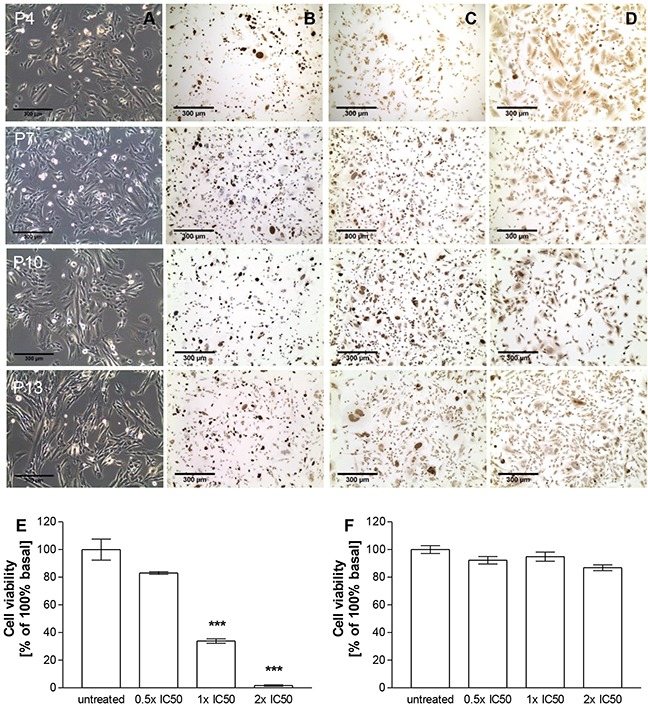
Bright-field pictures **A.** as well as Ki67 **B.**, SF-1 **C.** and 3βHSD **D.** stainings of MUC-1 cells in passages 4, 7, 10 and 13 *in vitro*. Treatment dependent inhibition of cell viability of NCI-H295R **E.** and MUC-1 **F.** upon addition of different concentrations of EDP-M *in vitro*. Stars represent statistical significance over untreated controls.

For the establishment of a novel *in vitro* ACC model we used explanted MUC-1 xenograft pieces for *in vitro* culturing. A first attempt failed after several passages due to massive contamination by murine fibroblasts. Thus, we initiated a second round of culturing involving continuous and highly specific murine and human fibroblast removal. The resulting multi-clonal cell suspension is now viable in passage 31. In these cultures, cross-contamination by murine fibroblasts could be excluded based on a universal primer probe assay using ApoE as specific target gene (Supplementary pdf 1). Representative pictures of passages 4, 7, 10 and 13 furthermore demonstrate specific stainings for Ki67, SF-1 as well as specific cytoplasmic 3βHSD expression for MUC-1 cells (Figure 5). Moreover, ELISA measurements revealed median cortisol levels of 0.8 ± 0.2 in cell culture supernatants MUC-1 cells during ongoing passaging (Figure 3B).

### Genetic characterization and comparison with commonly available ACC cell lines

Genetic characteristics of MUC-1 cells were investigated by PCR-Single-Locus Technology. These revealed a distinct short-tandem repeat (STR) profile for MUC-1 cells different from that of NCI-H295R and SW-13 cells (Table 1). Moreover, a sample with such STR profile is not reported in the online database of the DSMZ (German Collection of Microorganisms and Cell Cultures GmbH, https://www.dsmz.de). Thereby, MUC-1 cells were recently authenticated as a novel cell line of human origin (Supplementary pdf 2). Further genetic markers are summarized in Supplementary Table S1.

**Table 1:** Genetic characteristics of MUC-1 cells determined by PCR-Single-Locus Technology in comparison to the short tandem-repeat (STR) profiles of NCI-H295R and SW-13 as listed by ATCC

DNA marker	MUC-1	NCI-H295R	SW-13
AM	X	X	X
CSF1PO	12, 12	10, 12	11, 12
D13S317	9, 9	13	9
D16S539	11, 14	11	12
D5S818	11, 11	12	12
D7S820	8, 10	9, 12	8, 10
TH01	9.3 9.3	9, 3	7, 8
TPOX	8, 8	8	8
vWA	16, 17	17, 18	17, 19

### Evaluation of responsiveness to EDP-M treatment

As the patient had been treated with EDP-M followed by metastatic spread including the tumor sample, which had been used for the MUC-1 xenograft we sought to investigate the therapeutic responsiveness in comparison to NCI-H295R cells. These experiments identified NCI-H295R cells (Clone 1, Figure 5E) as sensitive to EDP-M (% of 100% basal; untreated: 100.0±7.6% vs. 0.5 × IC50: 83.1±1.0%, p<0.001; 1 × IC50: 33.8±1.6%, p<0.001; 2 × IC50: 1.7±0.5%, p<0.001), while MUC-1 cells (Figure 5F) were resistant against treatment with all concentrations (% of 100% basal; untreated: 100.0±2.8% vs. 0.5 × IC50: 92.2±2.7%, p>0.05; 1 × IC50: 94.8±3.3%, p>0.05; 2 × IC50: 86.8±2.1%, p>0.05).

## DISCUSSION

Only a few tumor models are available for ACC which can furthermore not reflect the high heterogeneity clinically observed for this tumor entity. However, even in those instances where different tumor models are available for a certain cancer type, it has been observed that cell lines diverge with extensive passaging *in vitro*, leading to a lack of clear correlation between therapeutic efficacy in cell-line based xenografts and clinical effectiveness. Key factors as invasive capabilities, genetic content, maintenance of a heterogeneous cell population, and the reliance on specific growth and survival pathways differ from the host and this process is until now only incompletely understood [15–17].

Our findings support this notion by demonstrating that different clones of the so far most commonly used tumor model for ACC significantly differ regarding tumor development, vascularization, proliferation, a marked decrease in engraftment potential following extensive passaging and most importantly in this context therapeutic responsiveness. This observation is endorsed by three independent working groups (reflected in Figure 1, Supplementary Figure S1 and by personal communication, Pierre Val, INSERM U1103, CNRS, Clermont Université, Clermont-Ferrand, France).

Keeping *in vivo* studies on the basis of patient-derived tissue-xenografts, separately from much more extensive passaging *in vitro*, has been already demonstrated to keep xenograft variability at bay [10]. Thus, to improve the predictive reliability of *in vivo* models patient-derived tumor xenografts (PDTXs) have been established for a wide range of cancer types and have been shown to maintain the original tumor architecture, histology, and gene expression in many instances [11, 18]. Accordingly, our workgroup aimed at the development of PDTX-models for endocrine tumors. Overall, some implanted and subsequently analyzed pieces remained vital and proliferating and retained specific patient characteristics in the murine host. However, in most instances these tumors lacked relevant growth properties (data not shown), limiting the applicability of PDTXs for preclinical therapeutic trials. In contrast to the majority of the established endocrine PDTXs, tissue-derived MUC-1 xenografts were characterized by the engraftment of large solid tumors and the maintenance of pathological and endocrine features comparable to that of the original patient tumor.

One of the most frequently cited reasons for the high failure rate of new therapeutic regimens in oncology is the lack of preclinical models reflecting patient heterogeneity [11, 15]. Initial characterization of MUC-1 xenografts and subsequent comparison with the commonly available tumor models for ACC revealed a molecular profile distinct from that of NCI-H295R, SW-13 and SJ-ACC3. In general, the histopathological diagnosis of adrenal tumors relies on a panel of different parameters: For the determination of the adrenocortical origin, the expression of SF-1 has emerged as the most valid marker [19] while for the discrimination of benign from malignant adrenocortical tumors the Ki67 index is of high importance. In addition to its value as prognostic marker, the Ki67 Index has been recently integrated in treatment flow charts for ACC patients and represents thereby an important determinant for ACC tumors [20, 21]. Moreover, EGF-R expression and furthermore membranous EGFR localization in immunohistochemical stainings has been reported to indicate a malignant phenotype of ACC [22].

Ki67 analyses of the different tumor models were mostly similar with slightly elevated indices for SW-13. SW-13-xenografts furthermore demonstrated an abrogation of both important markers, SF-1 and EGF-R, underlining the questionable value of SW-13 as tumor model for ACC. In contrast, NCI-H295R, SJ-ACC3 and MUC-1 derived tumors were positive for SF-1 and EGF-R. Interestingly, SJ-ACC3 displayed a highly significant overexpression of SF-1 RNA and protein, which is in accordance with previous reports on childhood adrenocortical tumors [23]. In contrast, MUC-1 tumors showed significantly elevated levels of EGF-R in comparison to the other tumor models.

Genetic analyses of known driver genes (*CTNNB1*, *TP53*, *CDKN2A*, *RB1* and *MEN1*) and of genes recently reported to be of importance in ACC (*ZNRF3*, *DAXX*, *TERT* and *MED12*) revealed in the original patient tumor (also representing passage 1) a somatic mutation in TP53 (a frameshift deletion: Hg19 positions: 7574003 on Chr 17: G is deleted) while the tumor was devoid of mutations in any of the other investigated genes (T91/L91 in [1]). SW-13 cells carry a homozygous *TP53* point mutation, NCI-H295R cells harbor a large deletion in the *TP53* locus and SJ-ACC3 cells display a *TP53* haplotype with G245C mutation as previously described for Li-Fraumeni syndrome [7, 8, 12]. NCI-H295R cells are furthermore known to have acquired an activating *CTNNB1* mutation [24], which has not been described for SW-13 and SJ-ACC3 yet. Our immunohistochemical analyses are in accordance with the underlying genetic phenotype for p53 and β-catenin. While we detected strong nucleo-cytoplasmic distribution of β-catenin in the NCI-H295R tumor model, the staining appeared weaker and of cytoplasmic localization in MUC-1. In contrast, while strong and nuclear p53 staining was evident in MUC-1, no specific immunopositivity was detectable in NCI-H295R. Overall, these findings indicate that MUC-1 and NCI-H295R might represent different patient clusters accordingly to recent classifications of adult ACC tumors [1, 25].

Of note, also MUC-1 cells maintained *in vitro* hormonal activity and specific phenotypical characteristics for ACC, which was furthermore proven stable over extensive passages. With regard to therapeutic responsiveness, MUC-1 cells demonstrated drug resistance against the clinical gold standard EDP-M, which was not observed for the commonly utilized tumor model NCI-H295R. This finding was not unexpected as the patient, from which MUC-1 was obtained, had received several cycles of EDP-M before development of metastases, while such treatment was not reported for NCI-H295R [8]. In this context it has to be mentioned, that even though superior over other chemotherapies, the overall response- rate of EDP-M is poor ranging from 20-50% with a median progression free survival of only five months [5, 26]. Consequently, to improve the identification of novel and/or second line therapies, preclinical experiments should include a tumor model reflecting the clinically low response rates to EDP-M. Following the same line, we were recently able to include MUC-1-xenografts for the first time in a therapeutic study with two different anti-IGF-1R inhibiting approaches. These experiments also indicated sub-group dependent differences in the expression of components of the IGF-system, as well as in therapeutic outcome versus NCI-H295R and SJ-ACC3, thereby also better reflecting clinical observations than with NCI-H295R alone [20, 27].

In summary, we herein report on the establishment of the first tumor model for ACC, which provides both a human cell line as well as a tissue-derived xenograft model. Moreover, we provide evidence that the implementation of a panel of NCI-H295R, SJ-ACC3 and MUC-1 might be helpful for a more successful clinical translation of novel therapeutic regimens for ACC in the future.

## MATERIALS AND METHODS

### Animals and tumor models

Female athymic NMRI *nu/nu* mice were purchased from Harlan Winkelmann (Borchen, Germany) and housed under pathogen-free conditions. Patient tumor derived pieces (approximately 2 × 2 mm) were implanted subcutaneously into the neck of individual mice. The number of mice for initial establishment and characterization was n=10 and n=4-20 for MUC-1-xenografts in different passages as outlined in detail below.

For NCI-H295 15×10^6^ and for SW-13 xenografts 13×10^6^ tumor cells in a volume of 200 μl PBS were inoculated while SJ-ACC3 tumors [12] were induced by direct tumor implantation of small tumor pieces into athymic nude mice. Before implantation of these 2 × 2 mm measuring tumor pieces appropriate samples of SJ-ACC3 tumors were transferred from liquid nitrogen to a 37°C water bath and tumor tissue was rinsed several times in medium 199 (Gibco Invitrogen, Darmstadt, Germany) supplemented with 1% penicillin/streptomycin. Therapeutic experiments were performed as previously described [13].

All experiments were carried out following protocols approved by the Regierung von Oberbayern and in accordance with the German guidelines for animal studies. Furthermore, studies including patient biomaterial were approved by the local ethical committee and patients provided written consent. Furthermore, experiments including patient biomaterial were approved by the local ethical committee.

### Pathological and immunohistochemical examination

Paraffin-embedded sections were rehydrated and incubated with blocking buffer containing 3 % BSA (Roche Diagnostics, Mannheim, Germany), 5 % goat or rabbit serum (Jackson ImmunoResearch Laboratories, West Grove, PA), and 0.5 % Tween 20 for 15 min. Immunohistochemical staining was performed using either monoclonal rabbit anti-human Ki67 (DCS, Hamburg, Germany; 1:200 in BB; Figures 1H and 1I, 2B and 2F, 4C and 5B), purified rat anti-mouse CD31 (Pharmingen, NJ, USA; 1:100 in BB; Figures 1E and 1F, 2D and 2H), monoclonal mouse anti-human SF-1 (Perseus Proteomics Inc., Tokyo, Japan; 1:100; Figures 2G, 4E, 5C), polyclonal rabbit anti-human SF-1, Novus Biological, Littleton, Colorado, USA; 1:200; Figure 2C), monoclonal rabbit anti-human EGF-receptor (Cell Signaling Technology, Danvers, USA; 1:100 in BB; Figure 4G) or polyclonal rabbit anti-mouse 3βHSD (provided by Anita Payne, University of Stanford, CA; Figure 5D), monoclonal mouse anti-human p53 (clone DO-7, Dako, Hamburg, Germany; 1:50 in BB; Figure 2I-L) and monoclonal mouse anti-human beta-Catenin (BD Transduction Laboratories, CA, USA; 1:500 in BB; Figure 2M-P) antibodies were used and incubated overnight at 4°C. After rinsing for 15 min in PBS, secondary antibody (for 3βHSD, Ki67, EGF-receptor and rabbit SF-1: goat anti-rabbit biotinylated IgG, Vector Laboratories, Burlingame, CA; for CD31: biotin-SP-conjugated AffiniPure goat anti-rat, Jackson Immuno Research Lab, CA; for mouse SF-1: ImmPRESS™ HRP Anti-Mouse IgG (Peroxidase) Polymer Detection Kit, Vector Laboratories; for p53 and beta-Catenin: biotinylated polyclonal rabbit anti-mouse, Dako, Hamburg, Germany) was applied for 30 min at room temperature. With the exception of a direct detection for the utilized SF-1 antibody complex, bound antibodies were visualized using the Vectastain ABC Kit (Vector Laboratories, Burlingame, CA, USA) followed by 3,3′-diaminobenzidine staining. For quantification 6 high power fields (HPF, 0.391 mm2, 400x magnification)/tumor were investigated and quantified for proliferation index (% Ki67 positive to negative cells).

The numbers of investigated samples for the different tumor models were n= 5-8 for Ki67, n=4-6 for SF-1, n=4 for EGF-R immunohistochemistry and n=4-7 for EGF-R and n=3-5 for SF-1 Real Time PCRs. The numbers of investigated samples for clones 1 and 2 of NCI-H295R were n= 5-7.

In vitro, 100 000 MUC-1 cells were seeded and PFA fixed on Falcon 4 well culture slides and incubated with the different antibodies as described for the tumor slides above.

### Cortisol measurements

Cortisol was measured in plasma samples of tumor bearing mice using the human Cortisol ELISA (n=6 for NCI-H295R and n=17 for MUC-1). Cortisol levels of cell culture medium from MUC-1 cells supernatants of passages 3, 4, 5, 6, 8, 9, 10, 12 and15 were analyzed and offset against a medium blank (in triplicate). Cortisol concentration was measured by a routine competitive automated chemiluminescence immunoassay (Liaison, Diasorin, Sallugia, Italy). According to the manufacturer, cross-reactivity is highest for prednisolone (12.6%), 11-desoxycortisol (3.0%) and corticosterone (3.5%) and negligible for other structurally related steroids. In the media used in this study, we confirmed LoQ at 0.5 μg/dL and within and between- assay variability at 2 μg/dL at 4.0% and 9.2%, respectively. For this study, all samples were analyzed on one day in one analytical run.

### Molecular investigation

SW-13, SJ-ACC3, NCI-H295R and MUC-1 tumors samples (n=4-7) were used for EGF-receptor Real-Time PCR analyses after RNA extraction (SV Total RNA Isolation system, Promega) and reverse transcription (RevertAid™ H Minus First Strand cDNA Synthesis Kit, Fermentas). For Real-Time PCR analyses we utilized the EvaGreen® reaction mix (Bio-Rad, Munich, Germany) in the Stratagene Mx3000PTM Cycler (Agilent Technologies, Waldbronn, Germany). Human primer catalogue numbers was #VHPS-10346 purchased from Biomol, Hamburg, Germany. For SF-1 Real-Time PCR analyses (n=3-5) human SF-1 Primer (forward: 5′-CAGCCTGGATTTGAAGTTCCT, reverse: 5′-CAGCATTTCGATGAGCAGGT) were used. Quantification was adjusted using the housekeeping gene GAPDH for human samples forward: 5′-AGC CTC CCG CTT CGC TCT CT-3′ and reverse: 5′-CCA GGC GCC CAA TAC GAC CA-3′.

### Cell culture

Both NCI-H295R and SW-13 cell lines were originally obtained from ATCC and again authenticated in February 2015. SW-13 cells were cultured in DMEM/F-12 medium (Dulbecco's Modified Eagle Medium, Gibco Invitrogen, Darmstadt, Germany) in a 5% CO2-95% air atmosphere at 37°C. Cell culture medium was supplemented with 1% penicillin/streptomycin and 10% FBS. NCI-H295R cells were cultured in a 1:1 mixture of DMEM and Ham's F-12 medium (DMEM-F12 and supplements, Gibco Invitrogen). The medium was supplemented with insulin (10 mg/ml), transferrin (5.5 mg/ml), and selenium (5 ng/ml) (ITS), penicillin (100 U/ml), streptomycin (100 μg/ml) and 2% ultroser G (Cytogen, Sinn, Germany).

For the establishment of a MUC-1 cell line a piece of the xenograft was minced into pieces smaller than 0.5 mm using a razor blade. The resulting suspension was transferred into a 50 ml Falcon tube and centrifuged for 5 minutes by 1000 rpm (Universal 30RF, Hettich, Tuttlingen, Germany). The supernatant was discarded followed by an incubation of the pellet with 1mg sterile collagenase II (Biochrom, Berlin, Germany) dissolved in PBS. After 50 minutes, FCS (Life Technologies, Carlsbad, California) to a concentration of 10% was added to inactivate the collagenase. Upon another centrifugation step at 1000 rpm for 5 minutes, the supernatant was discarded and the pellet re-suspended for seven minutes at room temperature in erythrocyte lysis buffer (2 volumes of lysis buffer onto 1 volume of cells). After a final centrifugation step, the cells were re-suspended, filtered through 70μm cell strainer and cultured in Advanced DMEM/F12 Medium (containing 10% FBS and 1% penicillin/streptomycin. The cells were split every 2 weeks and directly after cell culture establishment as well as after passages 2, 3, 4, 6, 9, 11, 13 and 14 a mouse and human fibroblast removal step was performed using the Anti-Fibroblast MicroBeads and Feeder Removal MicroBeads from (Miltenyi Biotec, Bergisch Gladbach, Germany) following the manufacturers instruction.

Successful clearance of murine fibroblasts from the target cell line was demonstrated by investigating genomic DNA by an in-house assay established by Eurofins (Ebersberg, Germany) and a Universal-Primer Probe-Assay using ApoE as specific target gene.

For cell viability assay (MTT) 40 000 NCI-H295R and 14 000 MUC-1 cells were seeded on a 96 well plate and cultivated for 24 hours either with EDP-M. For quantification of cell viability, a MTT assay (Sigma-Aldrich, Steinheim, Germany) was used following the manufacturer's protocols. Measurements were made in a SPECTRA microplate reader from Tecan (Crailsheim, Germany).

The tested concentrations were based on the individual IC50 (half-maximum inhibitory concentration) of each drug regarding cell proliferation: etoposide: 1.2μM; doxorubicin: 11μM; cisplatin: 9.6μM and mitotane: 15.9μM. In each case drugs were tested from their lowest to highest concentration (0.5x IC50, 1x IC50, 2x IC50) as a combination of etoposide, doxorubicin, cisplatin and mitotane (EDP-M).

### Genetic characterization

For detailed genetic characterization, genomic DNA of MUC-1 cells and the original patient tumor were extracted and a cell line authentication test was performed by Eurofins. Specifically, genetic characteristics were determined by PCR-single-locus technology. 21 independent PCR loci (Amelogenin, D3S1358, D1S1656, D6S1043, D13S317, Penta E, D16S539, D18S51, D2S1338, CSF1PO, Penta D, TH01, vWA, D21S11, D7S820, D5S818, TPOX, D8S1179, D12S391, D19S433 and FGA) were investigated (Promega, PowerPlex 21 PCR Kit). In parallel, positive and negative controls were carried out yielding correct results. The resulting sample with the DSMZ name CL151006_001 could not be verified in the online database of the DSMZ (German Collection of Microorganisms and Cell Cultures GmbH, http://www.dsmz.de/de/service/services-human-and-animal-cell-lines/online-stranalysis).

In a next step, genomic DNA of MUC-1 cells was investigated in more detail by Eurofins using the Argus-X12 PCR Kit for the analyses of nine independent PCR loci (Amelogenin, DXS8378, HPRTB, DXS7423, DXS7132, DXS10134, DXS10074, DXS10101, DXS10103, DXS10146, DXS10179, DXS10148 and DXS10135) including positive and negative controls.

### Statistical analysis

All results are expressed as mean ± SEM. Statistical significance was determined using one-way ANOVA with Bonferroni's multiple comparison test or the paired t-test (Prizm software, Houston, TX). Statistical significance was defined as p<0.05 and is denoted as stars (*, p<0.05; **, p<0.01; ***, p<0.001) in the Figures if not stated otherwise.

## SUPPLEMENTARY FIGURE AND TABLE


