# Supplementary material for: Targeting heterogeneity of adrenocortical carcinoma: Evaluation and extension of preclinical tumor models to improve clinical translation
Source: Oncotarget. 2016 Oct 15;7(48):79292–304. doi: 10.18632/oncotarget.12685 (PMC5346714; doi:10.18632/oncotarget.12685)
Supplement: Supplementary file 1 [file oncotarget-07-79292-s001.pdf]

Targeting heterogeneity of adrenocortical carcinoma: Evaluation and extension of preclinical tumor models to improve clinical translation

SUPPLEMENTARY FIGURE AND TABLE

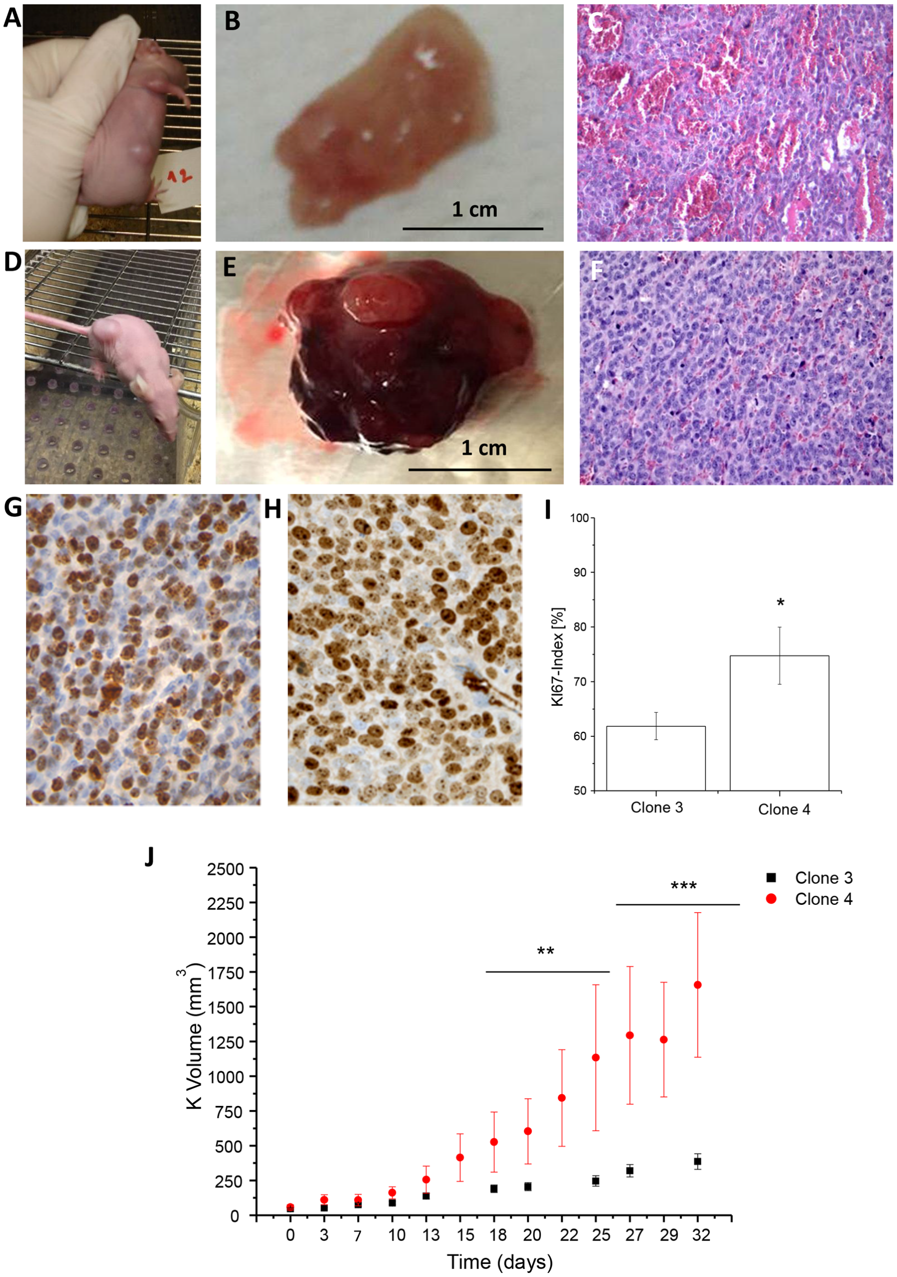

Supplementary Figure S1: Comparative features of xenografts obtained with different H295R clones. Pictures of representative athymic nude mice bearing H295R xenografts and H&E sections of clones 3 **A, B, C.** and 4 **D, E, F.** Representative Ki67 **G, H.** stainings as well as the quantification of proliferation index **I.** of the two tumoral clones. Statistical significance evaluated with Student's t- test: $p<0.05$, n=10). **J.** Differences in tumor growth rate of the H295R clones used for xenograft experiments. Stars denote significant differences starting from day 18 (**$p<0.01$; ***$p<0.001$, n=15 and n=6, clones 3 and 4 respectively). Animal studies were performed in compliance with an institutionally approved protocol (University of Florence) and according to the National Institutes of Health Guide for the Care and Use of Laboratory Animals.

eurofins Genomics

Eurofins Medigenomix GmbH, Anzinger Str. 7 a, D-85560 Ebersberg
Medizinische Klinik und Poliklinik IV
Endokrinologische Forschung
z. Hd. Dr. Constanze Hantel
Ludwig-Maximilians-Universität
Ziemssenstrasse 1
80336 München

Dr. Rainer Schubbert
Head of Operations Applied Genomics
and Head of Operational Excellence
Tel.: +49 8092 8289-277
Fax: +49 8092 8289-201
rainerschubbert@eurofins.com

29 January 2016

Subject: Examination for cross-contamination of murine DNA

**Sample Name:**	CL151006_001
**Sample Description:**	DNA
**Ordered by:**	Medizinische Klinik und Poliklinik IV / LMU München
**Sample Shipped from:**	Medizinische Klinik und Poliklinik IV / LMU München
**Received on:**	23.11.2015
**Packaging:**	tube
**Start/End of Analysis:**	03.02.2015 / 01.12.2015

Sample preparation, Method and References:
2 µL of the customer provided DNA sample were applied to one murine specific Real-Time-PCR assay (*18s* gene) to detect murine DNA. One positive and negative control were analysed simultaneously.

Test results:
The analyzed sample CL151006_001 showed no amplification product.

Conclusion:
The analyzed sample CL151006_001 showed no cross-contamination of murine DNA.

Yours Sincerely
Eurofins Medigenomix GmbH

Dr. Rainer Schubbert
Head of Operations Applied Genomics
and Head of Operational Excellence

Dr. biol. hum. Katharina Steinmann
Project Manager Applied Genomics

Our General Terms and Conditions apply exclusively and may be viewed or downloaded under eurofinsgenomics.com.
German law applies exclusively, with the exception of the UN-Convention Contracts for the International Sale of Goods.

Eurofins Medigenomix GmbH
Anzinger Straße 7 a
85560 Ebersberg
Germany

Tel.: +49 8092 8289-200
Fax: +49 8092 8289-201
Email: info-eu@eurofins.com
Web: eurofinsgenomics.com

Managing Directors: Dr. Peter Persigehl,
Bruno Poddevin, Ph.D.
Register Court Munich HRB 207710
VAT ID: DE815473648, Tax No.: 114/116/00167

HypoVereinsbank München
BSB: 700 202 70, Account No.: 273 50 40
IBAN: DE44 7002 0270 0002 7350 40
SWIFT: HYVE DEMMXXX

Supplementary Figure S2: Examination of MUC-1 cells for cross-contamination of murine DNA.

eurofins Genomics eurofins Forensik

DAkkS Deutsche Akkreditierungsstelle D-PL-18775-01-00

ISO 17025:2005 accredited

Klinikum der Universität München
Medizinische Klinik
Igor Shapiro
Ziemssenstr. 1
80336 München
Germany

28.10.2015

Certificate

Order

By order of Igor Shapiro (Klinikum der Universität München) we were requested to perform a cell line authentication test. Following samples were examined:

Our sample number	Client sample name
CL151006_001	unknown

Method:

DNA was isolated separately from the samples.
Genetic characteristics were determined by PCR-single-locus-technology. 21 independent PCR-systems Amelogenin, D3S1358, D1S1656, D6S1043, D13S317, Penta E, D16S539, D18S51, D2S1338, CSF1PO, Penta D, TH01, vWA, D21S11, D7S820, D5S818, TPOX, D8S1179, D12S391, D19S433 and FGA were investigated (Promega, PowerPlex 21 PCR Kit).
With the Argus-X12 PCR Kit nine (13) independent PCR-systems were analysed: Amelogenin, DXS8378, HPRTB, DXS7423, DXS7132, DXS10134, DXS10074, DXS10101, DXS10103, DXS10146, DXS10179, DXS10148 and DXS10135.

In parallel, positive and negative controls were carried out yielding correct results.

Supplementary Figure S3: Certificate of authentication of the novel MUC-1 cell line. (*Continued*)

eurofins | Genomics eurofins | Forensik

DAkkS Deutsche Akkreditierungsstelle D-PL-18779-01-00

ISO 17025:2005 accredited

Results:

DNA-System	DNA-criteria<br>unknown<br>CL151006_001
AM	X
DXS10074	8
DXS10079	20
DXS10101	30.2
DXS10103	19
DXS10134	37
DXS10135	28
DXS10146	29
DXS10148	18
DXS7132	15
DXS7423	14
DXS8378	11
HPRTB	11
D3S1358	16, 18
D1S1656	15, 15
D6S1043	12, 12
D13S317	9, 9
Penta E	7, 13
D16S539	11, 14
D18S51	14, 18
D2S1338	26, 26
CSF1PO	12, 12
Penta D	9, 9
TH01	9.3, 9.3
vWA	16, 17
D21S11	29, 29
D7S820	8, 10
D5S818	11, 11
TPOX	8, 8
D8S1179	8, 8
D12S391	19.3, 22
D19S433	13, 14
FGA	22, 23.2

Eurofins Medigenomix Forensik GmbH
Anzingerstr. 7a
D-85560 Ebersberg

Tel. + 49 (0) 8092 8289 260
Fax. + 49 (0) 8092 8289 190
www.eurofins.de/forensik-de
forensik@eurofins.com

Geschäftsführer
Dr. Peter Persigehl
Dr. Brigitte Obermaier
Dr. Bruno Poddevin

HRB 20 12 60
Amtsgericht München

Ust-Id Nr. DE 285161076
Steuernummer 114/116/00159

Supplementary Figure S3: (*Continued*) Certificate of authentication of the novel MUC-1 cell line. (*Continued*)

eurofins Genomics eurofins Forensik DAkkS ISO 17025:2005 accredited

Summary:

The following cell line was compared to the online database of the DSMZ (http://www.dsmz.de/de/service/services-human-and-animal-cell-lines/online-str-analysis.html):

Our sample number	Client sample name	DSMZ name
CL151006_001	unknown	Not detected in the DSMZ

The sample CL151006_001 is of human and potentially male origin. The loss of the Y chromosome was proven by X-chromosomal markers showing a hemizygous profile.

Dr. Burkhard Rolf
Director Forensic Services

Dr. Kerstin Knoop
Project Manager DNA-Forensics

Eurofins Medigenomix Forensik GmbH carries out all analyses with greatest care and on the basis of state of the art scientific knowledge. All results solely refer to the analysed samples. Our expert's reports must not be duplicated in extracts without consent of Eurofins Medigenomix Forensik GmbH.
Cell_line-certificate_eng_V03_141211

Vorlage_Verwandtschaftsanalyse_EUROFINS_v02_121127

Eurofins Medigenomix	Tel. + 49 (0) 8092 8289 260	Geschäftsführer	HRB 20 12 60	Ust-Id Nr. DE 285161076
Forensik GmbH	Fax. + 49 (0) 8092 8289 190	Dr. Peter Persigehl	Amtsgericht München	Steuernummer 114/116/00159
Anzingerstr. 7a	www.eurofins.de/forensik-de	Dr. Brigitte Obermaier		
D-85560 Ebersberg	forensik@eurofins.com	Dr. Bruno Poddevin		

Supplementary Figure S3: (*Continued*) Certificate of authentication of the novel MUC-1 cell line.

Supplementary Table S1: Detailed cell line authentication profile of MUC-1 cells

DNA-System	DNA-Criteria
AM	X
DXS10074	8
DXS10079	20
DXS10101	30.2
DXS10103	19
DXS10134	37
DXS10135	28
DXS10146	29
DXS10148	18
DXS7132	15
DXS7423	14
DXS8378	11
HPRTB	11
D3S1358	16, 18
D1S1656	15, 15
D6S1043	12, 12
D13S317	9, 9
Penta E	7, 13
D16S539	11, 14
D18S51	14, 18
D2S1338	26, 26
CSF1PO	12, 12
Penta D	9, 9
TH01	9.3 9.3
vWA	16, 17
D21S11	29, 29
D7S820	8, 10
D5S818	11, 11
TPOX	8, 8
D8S1179	8, 8
D12S391	19.3, 22
D19S433	13, 14
FGA	22, 23.2

Genetic characteristics determined by PCR-Single-Locus Technology.
